# Monoalkylated Epigallocatechin-3-gallate (C18-EGCG) as Novel Lipophilic EGCG Derivative: Characterization and Antioxidant Evaluation

**DOI:** 10.3390/antiox9030208

**Published:** 2020-03-03

**Authors:** Cristina Minnelli, Roberta Galeazzi, Emiliano Laudadio, Adolfo Amici, Dario Rusciano, Tatiana Armeni, Mattia Cantarini, Pierluigi Stipa, Giovanna Mobbili

**Affiliations:** 1 Department of Life and Environmental Sciences, Università Politecnica delle Marche, via Brecce Bianche, 60131 Ancona, Italy; c.minnelli@staff.univpm.it (C.M.); r.galeazzi@staff.univpm.it (R.G.); m.cantarini@pm.univpm.it (M.C.); 2 Department S.I.M.A.U., Università Politecnica delle Marche, via Brecce Bianche, 60131 Ancona, Italy; e.laudadio@staff.univpm.it (E.L.); p.stipa@staff.univpm.it (P.S.); 3 Department of Clinical Sciences, Section of Biochemistry, Biology and Physics, Università Politecnica delle Marche, via Brecce Bianche, 60131 Ancona, Italy; a.amici@staff.univpm.it (A.A.); t.armeni@staff.univpm.it (T.A.); 4 Sooft Italy SpA, 95100 Catania, Italy; dario.rusciano@sooft.it

**Keywords:** epigallocatechin-3-gallate, antioxidants, oxidative stress, molecular dynamics (MD) simulation, free radicals

## Abstract

Epigallocatechin-3-gallate (EGCG) has the highest antioxidant activity compared to the others catechins of green tea. However, the beneficial effects are mainly limited by its poor membrane permeability. A derivatization strategy to increase the EGCG interaction with lipid membranes is considered as one feasible approach to expand its application in lipophilic media, in particular the cellular absorption. At this purpose the hydrophilic EGCG was modified by inserting an aliphatic C18 chain linked to the gallate ring by an ethereal bond, the structure determined by NMR (Nuclear Magnetic Resonance) and confirmed by Density Functional Theory (DFT) calculations. The *in vitro* antioxidant activity of the mono-alkylated EGCG (C18-EGCG) was studied by the DPPH and Thiobarbituric Acid Reactive Substances (TBARS) assays, and its ability to protect cells towards oxidative stress was evaluated in Adult Retinal Pigmented Epithelium (ARPE-19) cells. Molecular Dynamics (MD) simulation and liposomal/buffer partition were used to study the interaction of the modified and unmodified antioxidants with a cell membrane model: the combined experimental-*in silico* approach shed light on the higher affinity of C18-EGCG toward lipid bilayer. Although the DPPH assay stated that the functionalization decreases the EGCG activity against free radicals, from cellular experiments it resulted that the lipid moiety increases the antioxidant protection of the new lipophilic derivative.

## 1. Introduction

**Figure 1:**
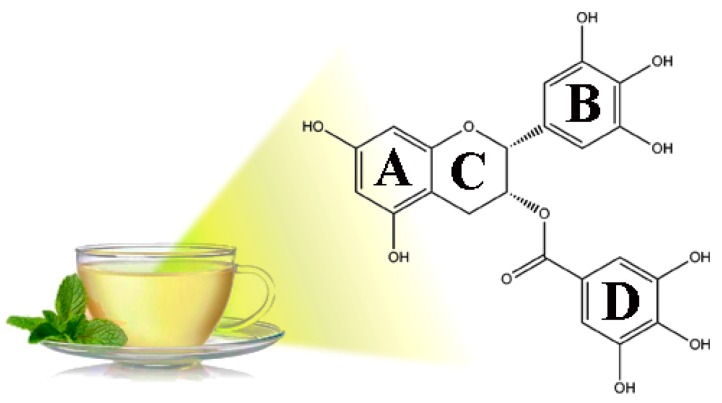
Structure of epigallocatechin-3-gallate, a green tea polyphenol.

Epigallocatechin-3-gallate (EGCG), a polyphenolic catechin from green tea, is a well-known molecule active in age-related illnesses where oxidative stress plays a key role, including cataract, Dry Eye Syndrome (DES) and Age-Related Macular Degeneration (AMD) [1,2,3]. The EGCG structure presents a benzenediol ring (label A) joined to a tetrahydropyran moiety (C), a pyrogallol ring (B) and a galloyl ring (D) (Figure 1). 

**Scheme 1:**
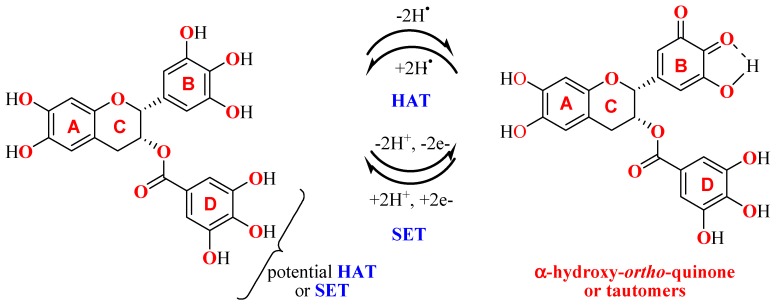
α-hydroxy-*ortho*-quinone formation from EGCG by Hydrogen Atom Transfer (HAT) or Single Electron Transfer Mechanisms (SET).

Multiple mechanisms contribute to the overall antioxidant effect: radical scavenging activity [4,5], chelation of transition metals, which are natural substrate responsible of the Fenton or Fenton-like reactions, [6] and ability to increase the expression of antioxidant enzymes [7]. The antioxidant activity mainly arises from the reactivity of the hydroxy-substituted B and D aromatic rings which can neutralize free radicals through Single Electron Transfer Mechanisms (SET) and/or Hydrogen Atom Transfer (HAT). The free radical quenching mechanism by HAT or SET produces a phenoxy radical, further stabilized by intramolecular hydrogen bonding, which tends to be converted in α-hydroxy-*ortho*-quinone (Scheme 1) [8]. Both galloyl and pyrogalloyl moieties can potentially undergo HAT thus eliciting the high antioxidant activity of EGCG. 

EGCG can therefore act as chain-breaking antioxidant [9] through the inhibition of lipid peroxidation, representing a potential source of detrimental ROS in degenerative diseases [6].

EGCG beneficial effects are however counteracted by its poor membrane permeability, rapid metabolism and chemical instability in physiological environment [10,11]. The stabilization of EGCG can be obtained by different approaches including the employment of reducing agents, structural modification and/or drug delivery systems [12,13,14]. Among these, the application of derivatization strategies designed to increase the molecule hydrophobicity and the interaction with cellular membrane [15,16,17,18,19,20] is considered as a feasible approach to improve the activity of bioactive molecules. 

In order to obtain a membrane-targeting antioxidant, the hydrophilic structure of EGCG has been modified by inserting an aliphatic C18 chain linked to the D-ring by an ethereal bond. Similar structural modifications have been reported for the EGCG structure, in particular concerning the synthesis of ester derivatives [21]; no ether functionalization has been reported yet. Thus, we decided to go further in this direction, considering that the ether linkage is chemically more resistant to hydrolisys. The position of the octadecyl group has been determined by NMR (Nuclear Magnetic Resonance) and supported by DFT calculations. The increased affinity of the derivative for a lipid bilayer was studied by an integrated *in silico*/experimental approach in a liposome model membrane system. The antioxidant activity of the C18-EGCG ethereal derivative was studied *in vitro* by DPPH and by measuring the amount of Thiobarbituric Acid Reactive Substances (TBARS) produced in lipid peroxidation experiments, performed on artificial lipid bilayers systems; then, as a part of a research project focused on the development of antioxidants to be employed in AMD, its ability to contrast H_2_O_2_-induced oxidative stress was studied in Adult Retinal Pigmented Epithelium (ARPE-19) cells. 

## 2. Materials and Methods 

### 2.1. Materials

Commercial phosphatidylcholine from egg yolk (PC), the α,α-diphenyl-β-picrylhydrazyl (DPPH) and the azocompound 2,2-azobis(2-amidinopropane hydrochloride) (AAPH) were obtained from SigmaAldrich Co. (Stenheim, Germany). Epigallocatechin-3-gallate (EGCG) was purchased from Cayman Chemical Company (Ann Arbor, MI, USA). All the other reagents and chemicals were of analytical grade and were used without further purification. In order to prevent metal contamination, all the solutions were prepared in ultrapure MilliQ water. All material and reagents employed in the synthetic procedures were purchased from SigmaAldrich Co. (Stenheim, Germany) unless otherwise stated and used without purification. The solvents used were analytically pure. All synthesis have been performed in an argon atmosphere. TLC were carried out on aluminium sheets precoated with silica gel 60 F254 (Merck), while for column chromatography silica gel 60 (230–400 mesh) has been employed. Electrospray ionization mass spectra were obtained with a Finnigan Navigator single-quadrupole mass spectrometer, cone voltage 25 V and capillary voltage 2.5 kV, injecting samples dissolved in methanol. ^1^H and ^13^C NMR spectra were recorded at 400 and 100 MHz respectively, on a Varian Gemini 200 spectrometer, using Acetone-d6 as solvents. Chemical shifts (δ) are reported in ppm relative to TMS and coupling constants (J) in Hz. Adult human retinal pigment epithelial (ARPE-19) cells were purchased from American type Culture Collection (ATCC, CRL-2302). All cell culture reagents were purchased from Euroclone (Euroclone, Italy). All other chemicals and buffer components were of analytical grade preparations.

### 2.2. Synthetic Procedure

To a stirred solution of EGCG (700 mg, 1.52 mmol) and K_2_CO_3_ (210 mg, 1.52 mmol) in anhydrous DMF (10 mL), octadecyl iodide in cyclohexane (578 mg, 1.52 mmol) was slowly added in 1 h in an Ar atmosphere. The solution was then heated at 40 °C for 8 h, and after the addition of MQ water/DMF mixture (10:1 *v/v*, water: DMF) the crude product was lyophilized. The purification was performed by silica gel column chromatography (cyclohexane-EtOAc, 75:25) to afford C18-EGCG (7.6 g, 53%) as a brown solid. 

^1^H NMR (Acetone-*d*_6_, 400 MHz): δ = 0.87 (t, *J* = 7.4 Hz, 3H), 1.20–1.35 (m, 30H), 1.35–1.45 (m, 1H), 1.62–1.75 (m, 1H), 2.97 (dd, *J* = 2.4 Hz, 13.9 Hz, 1H), 3.03 (dd, *J* = 3.9 Hz, *J* = 13.9 Hz, 1H), 4.09 (t, *J* = 5.5, 2H), 5.07 (s, 1H), 5.42 (s, 1H), 6.03 (m, 2H), 6.67 (s, 2H), 7.05 (s, 2H).

^13^C NMR (Acetone-*d*_6_, 100 MHz): δ = 13.5, 22.4, 25.7, 30.8, 31.7, 62.3, 69.3, 70.2, 73.3, 78.3, 95.9, 96.5, 98.9, 106.8, 109.9, 126.2, 129.6, 130.5, 133.5, 139.6, 146.6, 151.4, 157.1, 157.5, 157.8, 166.3.

ESI-MS: (*m*/*z*) 823 [M + CF3COO^−^].

### 2.3. 2,2-Diphenyl-1-picrylhydrazyl (DPPH) Assay

In the DPPH radical scavenging assay appropriate aliquots of antioxidant were mixed with DPPH in methanol (antioxidant 25 µM, DPPH 100 µM, FC); the reaction mixtures were shaken and then incubated for 30 min in the dark. The absorbances of the reaction mixtures and of a DPPH solution used as a negative control were then measured at 517 nm with a spectrophotometer (BioTek Synergy HT MicroPlate Reader Spectrophotometer) against methanol as blank. The inhibition percentage of antioxidants was calculated according to the following equation:

Inhibition ratio (%) = [(A_control_ − A_sample_)/A_control_] × 100

where A_control_ is the absorbance of the control obtained by adding to DPPH methanol solution a methanol aliquot equal to the antioxidant solution volume added, and A_sample_ is the absorbance of the reaction solution at 30 min. All the experiments were repeated at least three times and measurements were run in triplicate.

### 2.4. Computational Methods

#### 2.4.1. Density Functional Theory Calculations

Gaussian 09 package of programs [22] has been used to carry out all quantum mechanical calculations, at Becke-Lee-Parr hybrid exchange correlation three-parameter functional (B3LYP) [23] level, 6-311G+(d,p) basis set and PCM continuum model [24]. The method’s reliability has been extensively proven [25,26,27]. Geometry optimization and vibrational frequencies were calculated for all the species in aqueous solution. Vibrational analysis showed that the optimized structures were in accordance with the minimum points on the potential energy surface (i.e., absence of imaginary frequencies). 

#### 2.4.2. Molecular Dynamics Simulation

A mixed composition bilayer model containing 256 lipids divided in two eaftlets was assembled. The lipid composition was considered with the aim to mimic the egg-PC (from egg yolk, type XVI-E, ≥99% (TLC), lyophilized powder prepared by a modification of the procedure of Singleton et al. Sigma-Aldrich) [28], introducing 110 POPC; 38 PLPC; 28 SOPC; 28 SLPC; 26 DOPC; 18 DSPC; 8 DPPC. The membrane builder tool (Charmm-GUI, availble on line at www.charmm-gui.org; accessed on January 2019) was used for the starting pure egg−PC model that after minimization, was stabilized by a 10 ns equilibration by NPT MD simulation [29].

Then, pre-equilibrated PC lipids were added randomly substituted adding six C18-EGCG molecules in order to reproduce the experimental samples’percentage. The box dimensions were 9.34 × 9.36 × 8.62 nm, and the simulation model was hydrated by adding 11241 water molecules. AMBER force field was used for all the MD simulations treating membrane with lipid parameter sets [30] (GROMACS 5.0.4 suite of programs) [31]. The bilayer energy was minimized in periodic box conditions (PBC), using a neighbor searching grid type. The cut-off distance for the short-range neighbor list was set to 1.4 nm. Electrostatic interactions were taken into account by fast smooth particle-mesh Ewald algorithm [32,33,34,35] considering a 1.4 nm distance for the Coulomb cutoff.

The efficiency and precision of this method is well known in the evaluation of long-range electrostatic interactions in macromolecular systems [36,37]. On the minimized structures, 100 ns atomistic MD simulation was carried in the isothermal−isobaric (NPT) ensemble at 1 atm and 310 K (37 °C) [38]. A 2 ns of annealing simulations increased gradually the temperature up to 310 K in and then the atomic velocities were generated; a weak temperature coupling (Berendsen thermostat), with a time constant of 1 ps, was applied to maintain the reference temperature (310 K) throughout the whole run; no water was observed inside the bilayer. In the production stage, 100 ns MD runs have been carried out for the egg−PC bilayer/antioxidant bilayer. 

The leap-frog stochastic dynamics integrator was used as the main run control option with a time step of 0.002 ps. The first 2 ns MD simulation for the lipid system was carried out in the NVT ensemble using a Langevin thermostat, whereas the subsequent nanoseconds were carried out in the NPT ensemble (T = 310 K, P = 1 atm) using semi-isotropic pressure coupling and Berendsen thermostat. A 0.5 ps time constant for coupling and 4.5 × 10^−5^ bar^−1^ optimal compressibility for water were in order to keep the best pressure control. The steady state achievement was monitored for all the simulated models through the molecular root-mean-square deviation (rmsd) values vs. time [39]. Deviation was calculated as displacement with respect to the starting minimized structure. 

The fluctuations of the C18-EGCG molecules and their dynamical behaviour has been analized focusing on the the orientation of EGCG-C18 head groups in the PC lipid system during MD simulations, and the mass density profile of the C18-EGCG aromatic groups was computed. The subsequent analysis of the bilayer properties was performed on the final 20 ns of the MD trajectories, that for all the systems correspond to the steady-state condition as results from RMSD plots. The analysis of the simulations’ trajectories was performed by means of the VMD and CHIMERA software [40,41]. 

### 2.5. Preparation of Large Unilamellar Vesicles (LUVs)

Unilamellar liposomes (LUVs) were prepared by the “thin film hydration” method. Appropriate amounts of chloroform solutions of L-α-phosphatidylcholine (100 mg mL^−1^) and, where required, of tested antioxidant compound (10 mM) were mixed in a round bottom flask and dried under nitrogen followed by high vacuum for 2 h. The film was added with an appropriate amount of 20 mM PB (pH 7.4), and vortexed for 5 min to obtain a opalescent aqueous suspension (3 mM PC, 0.06 mM antioxidant, FC) of multilamellar vesicles (MLV). After incubating overnight to swell and stabilize, the vesicle suspensions were extruded 21 times through two polycarbonate membranes (Avanti Polar Lipids Inc., Alabaster, AL, pore size 200nm) using the Mini Extruder from Avantipolar Lipids Inc. All extrusions were performed at room temperature. A homogeneous population of LUV (Large Unilamellar Vesicles) was obtained, as assessed by Dynamic Light Scattering [42]. The liposome suspensions were used both in the AAPH-peroxidation experiments and in the studies of antioxidant interaction with liposome as cell membrane models.

### 2.6. Peroxidation of LUVs

The lipid peroxidation was initiated into PC liposomal membranes by peroxyl radicals generated at a constant rate through thermal degradation of the hydrophilic azocompound AAPH [43]. Then, 300 µL of each LUV dispersion with and without antioxidant were added with 25 µL AAPH 65 mM (5 mM FC) and incubated for 2 h at 37 °C; in order to obtain non-oxidized samples, and 300 µL of each dispersion were added with 25 µL of PBS and 10 µL of 10 mM ethanolic BHT. After the incubation time, the PC peroxidation was stopped by adding 10 µL of 10 mM ethanolic BHT to avoid further sample oxidation while processing. 900 µL of TBA–TCA–HCl (0.375% w/v TBA, 15% w/v TCA, 0.2 M HCl) were then added and the samples were heated for 15 min at 95 °C, cooled and then centrifugated at 1400 g for 10 min. The absorbance of the pink MDA-TBA adduct developed upon heating was measured in the supernatant at 532 nm for the determination of the aldehydic breakdown products of lipid peroxidation (TBARS). The antioxidant activity of the studied compounds was expressed as % inhibition according to the following equation:

% inhibition = (1 − ΔA_sample_ / ΔA_PC_) × 100,

where ΔA_sample_ is the difference of the absorbance between the oxidized and non-oxidized sample containing the tested antioxidant compound and ΔA_PC_ is the difference of the absorbance between the oxidized and non-oxidized PC. All the experiments were repeated at least three times and measurements were run in triplicate.

### 2.7. Liposomal/Buffer Partitioning

The liposomal/buffer partitioning was stadied by adding appropriate amounts of methanol solutions of EGCG and C18-EGCG (0.1, 0.25, and 0.5 mM, antioxidant FC) to 1 mL of PC LUV in PBS (6 mM, lipid FC). The liposomal emulsions were vortexed and incubated for 20 min at 37 °C. Next, the non-encapsulated antioxidants were separated from liposomes by size exclusion chromatography. After preconditioning with PBS, 1 mL of EGCG or C18-EGCG liposomal suspension were gently added on the top of the packed Sephadex G-50 resin coloumn and centrifuged at 500g for 10 min. Free EGCG and C18-EGCG were also used as control. After collecting the eluates, the Stewart assay was carried out to determine the lipid content in the liposome preparation after gel filtration. To evaluate the amount of EGCG and C18-EGCG interacting with liposome membrane, 150 μL samples of purified and unpurified liposomes were lysed by addition of Triton X-100 to a final concentration of 1% (*v/v*) in order to achieve the complete release of the antioxidant. After lysis, the polyphenol concentration was estimated by Folin-Ciocalteu assay [44]: 10% Folin-Ciocalteu reagent (0.15 mL) was added to each sample (0.05 mL) into a 96-well microplate and shaken. After 10 min, 0.1 mL of a 7.5% sodium carbonate solution was added followed by incubation in the dark for 1 h at room temperature. The absorbance was then measured at 765 nm on a BioTek Synergy HT MicroPlate Reader Spectrophotometer using an appropriate blank. Calibration curve were plotted using EGCG and C18-EGCG. All the experiments were repeated at least three times and measurements were run in triplicate. 

### 2.8. Cell Treatment

ARPE19 cells were routinely maintained in 25 cm^2^ flasks in complete DMEM/F12 medium at 37 °C, 5% CO_2_ and 95% relative humidity. The complete DMEM/F12 medium was prepared by adding 10% (*v/v*) fetal bovine serum (FBS), 2 mM glutamine and 100 U/ml penicillin-streptomycin. Culture medium was changed every 2 days until cells grew to 90% confluence. The cell cultures were detached by trypsinization with 0.5% trypsin in PBS containing 0.025% EDTA. ARPE19 cells were seeded in 96-well plates at 1 × 10^4^/well to reach 60% of confluence at 24 h. The cells were treated for 48 h with increasing concentrations (10, 20, 40, 60, 80, 120, 160, 240, 320 and 640 µM) of EGCG and C18-EGCG. Dose-dependent curves were therefore generated for the cytotoxic studies. The 50% inhibiting concentration (IC50) were determined by nonlinear regression analysis with a three-parameter fit by utilizing a SigmaPlot 12.0 Software. In the evaluation of protection of oxidative stress induced-cell death, EGCG and C18-EGCG were tested in the 20–80 µM concentration range. After 24 h of incubation, the cells were washed twice with PBS and exposed to 6 mM for 24 h. The H_2_O_2_ concentration able to induce of about 50% of cell death was determined by previous MTT viability assay (data not shown).

### 2.9. Cell Viability (MTT Assay)

To evaluate the cell viability, the 3-(4,5-dimethylthiazol-2-yl)-2,5-diphenyltetrazolium bromide (MTT) assay was used [45]. Before the assay, the medium of each well was substituted with DMEM/F12 supplemented with MTT at a final concentration of 0.1 mg mL^-1^. After incubation of samples for 3 h at 37 °C in 5% CO_2_ atmosphere formazan crystals were formed which were then solubilize by adding 0.4 mL of DMSO to each well. Absorbance was read at 570 nm using the extraction buffer as blank. The optical density in untreated cells was considered as 100% viability. The relative cell viability (%) was calculated as (OD570 of treated cells/OD570 of untreated cells) × 100. Each experiment was performed at least five times in triplicate. 

### 2.10. Statistical Analyses

Data are presented as mean ± S.D (Standard Deviation). Statistical analyses were performed by using Student’s t-test. *p*-values ≤ 0.05 were considered statistically significant, *p*-values ≤ 0.01 and *p*-values ≤ 0.001 were considered highly significant.

## 3. Results and Discussion

### 3.1. Synthesis and Structural Elucidation of C18-EGCG

**Scheme 2:**
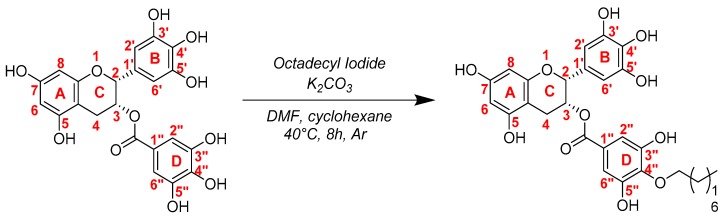
Synthesis of C18-EGCG.

With the aim to obtaini a more lipophilic EGCG derivative with an improved affinity for a lipid bilayer and other oxidation-susceptible cellular sites, the molecular skeleton of the natural antioxidant EGCG was modified by the introduction of a C-18 hydrocarbon chain; such an aliphatic tail has been bound by an ether linkage employing the Williamson reaction (Scheme 2) and the structure of the main mono-alkylated product has been confirmed by nuclear magnetic resonance (NMR) specroscopy and mass spectrometry.

**Figure 2:**
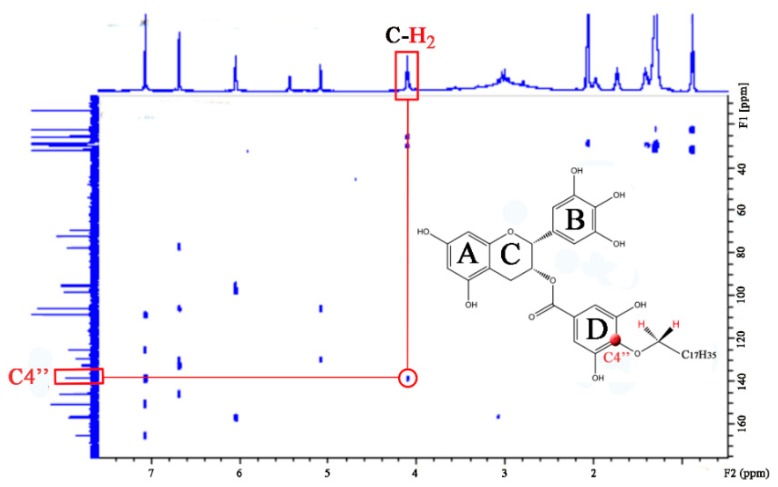
HMBC (Heteronuclear Multiple-Bond Connectivity Experiment). Cross-peak between C4” and methylenic protons of C18 chain directly bound to the oxygen in C4”.

The ^1^H and ^13^C NMR assignments were performed by means of Heteronuclear Multiple-Bond Correlation method (HMBC) and by comparison with the data previously reported [46]. The ^1^H NMR spectrum of C18-EGCG was very close to that of EGCG, except for the protons attributable to the C18-chain (Table 1) while the ^13^C NMR of the alkylated derivate shows significative differences in the chemical shifts of the carbons at positions 1″-, 3″-, 4″- and 5″-, suggesting the alkylation of the D-ring (Table 2). In addition, the absence of separation between H2″ and H6″ signals in the ^1^H NMR spectrum was interpreted as a consequence of the EGCG alkylation in 4″ position. In the HMBC experiment, a cross-peak between C-4″ and the methylenoxy protons of C18 chain confirmed this hypothesis (Figure 2). The 4″- selective alkylation of EGCG could be attributed to the stabilizing effect of the carbonyl group with respect to the phenolate anion at the 4’-position. 

This hypothesis is also supported by DFT calculations, by which the relative energy and stability of the possible eight oxyanions on the four EGCG rings have been computed in order to compare the acidity of their corresponding phenolic form (Table 3). From the data reported, EGCG-oxyanion in position 4O″ of ring D appears to be the most stable with respect to all the other oxyanions, thus suggesting the stronger acidity of the correspoindg hydroxyl proton; in addition, the other oxyanions are much higher in energy and thus not significatively favoured. This firmly supports the hypothesis that this position should represent the most suitable for alkylation.

### 3.2. Reactions with DPPH Free Radical

**Figure 3:**
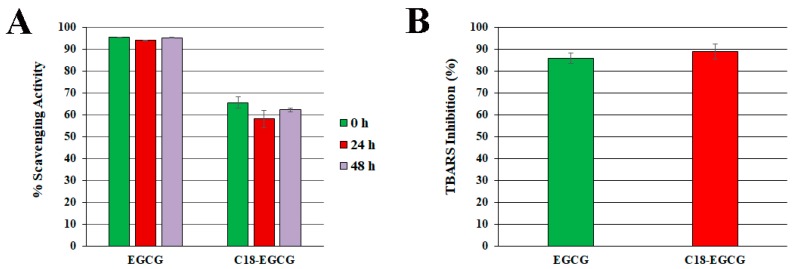
(**A**) DPPH assay of antioxidant activity of EGCG and C18-EGCG and at different time of analyses (0, 24 and 48 h). The values in the figures are expressed as the means ± SD; n = 7 independent experiments. (**B**) % Inhibition of TBARS formation in PC (3 mM) liposomes peroxidation by EGCG and C18-EGCG (0.05 mM) induced by thermal decomposition (37 °C, 1 h) of AAPH (5 mM). The values in the figures are reported as the means ± SD; n=5 independent experiments.

The DPPH (2,2-diphenyl-1-picrylhydrazyl) assay was used to compare the scavenging activity of C18-EGCG and the unmodified EGCG toward free radicals at different time intervals (0, 24 and 48 h); the results are presented in Figure 3A as percentage inhibition. Both compounds tested showed the ability to quench DPPH, and this activity did not change with time in our experiments: in this case, EGCG was found to be the most effective (percentage inhibition, ca. 95%), while the presence of C18 carbon chain in its lipophilic analogue determined a reduction in the antioxidant activity of about 30% (percentage inhibition ca. 65%). This behavior could be attributed to the functionalization in the 4″ D-ring position, which decreases the antioxidant ability (DPPH reduction); in addition, the unavailability of the OH-proton in 4″ position in C18-EGCG hampers the consequent oxidation of the other two hydroxyls present in the D-ring to the corresponding ortho-quinone moiety, in analogy to the mechanism described in Scheme 1 for ring B.

### 3.3. Protection against Free Radical-Mediated Lipid Peroxidation 

The antioxidant activity of C18-EGCG and EGCG was also evaluated by inducing by AAPH lipid peroxidation in PC liposomes and measuring the percentage inhibition of aldehydic breakdown products (TBARS) formation. AAPH was used to generate the initiating radicals in the aqueous phase: its thermolysis produces tertiary carbon centered radicals, which rapidly react with the molecular oxygen present in the reaction medium generating a constant flux of peroxyl radicals, responsible of lipid peroxidation through an hydrogen abstraction mechanism. In this model, an antioxidant can undergo two different mechanism: (1) direct reaction with the hydrophilic C-centered radicals generated from AAPH degradation in the aqueous compartment; (2) inhibition of the lipid peroxidation cascade reaction in the lipid phase, through a chain-breaking effect involving lipid peroxyl radical quenching by HAT mechanism. As showed in Figure 3b, no significative differences were observed in the ability to inhibit lipid peroxidation of both tested derivatives (percentage inhibition, 86 and 89% for EGCG and C18-EGCG respectively) in contrast with what observed in the DPPH assay.

### 3.4. Studies on Interaction of EGCG and C18-EGCG with Liposome Cell Membrane Models

In order to better evaluate the impact of the hydrophobicity on the antioxidant activity exhibited by EGCG and C18-EGCG in the TBARS assay, the interaction of both antioxidants with a liposome cell membrane model has been studied by an integrated *in silico*/experimental approach. 

#### Affinity of EGCG and C18-EGCG for Lipid Bilayers 

The study of the liposomal/buffer partition represents an important approach to evaluate the affinity of a compound toward a lipid bilayer. The amounts of EGCG and C18-EGCG incorporated into PC LUV liposomes, employed as a cell membrane model, were calculated starting from different polyphenol concentrations corresponding to 0.1, 0.25 and 0.5 mM. 

**Figure 4:**
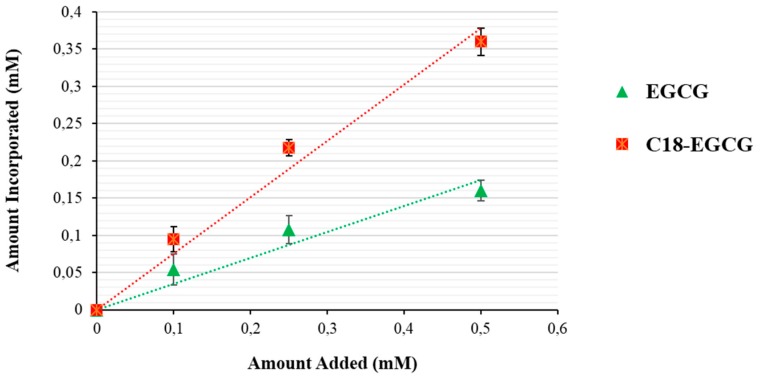
Methanol solutions of EGCG (green) and C18-EGCG (red) (0.1 mL) were added to liposomal suspensions (0.9 mL). Folin-Ciocalteu method was used to determine the antioxidant amount incorporated into liposomes. The values in the figures are expressed as the means ± SD; n=5 independent experiments.

The results, presented in Figure 4, show a linear relationship between the encapsulated amounts of the compounds and the amount added to the liposomal solution in the range tested. The concentration of polyphenol incorporated in the liposomes was higher for C18-EGCG than for EGCG accounting for a larger affinity of the former derivative toward the liposomal lipid bilayer. The slopes of the two linear functions can be correlated with the affinity factors of the antioxidants for the liposomal membrane, and potentially for the cell lipid bilayer [47]. At this regard, the hydrocarbon chain introduced determines for the C18-EGCG an affinity factor of 0.76 (R^2^ = 0.98) which is more than twice that of EGCG (0.35, R^2^ = 0.93). It is noteworthy that at a lipid/antioxidant ratio of 50 as employed in the TBARS assayes, C18-EGCG is a constituent of the PC bilayer while EGCG is incorporated for less than 50% (molar ratio).

In addition, the employement of EGCG concentrations above 0.5 mM had the effect to form large aggregates precipitating within the sample tube (data not shown). This appears to be in agreement with previous studies, in which emerged the propensity of EGCG to increase precipitation phenomena in the vesicle suspensions [48,49,50]; interestingly, the suspension mixed with C18-EGCG did not yield any precipitate. 

### 3.5. Structural Insight of C18-EGCG inside Lipid Bilayer: In Silico Studies

**Figure 5:**
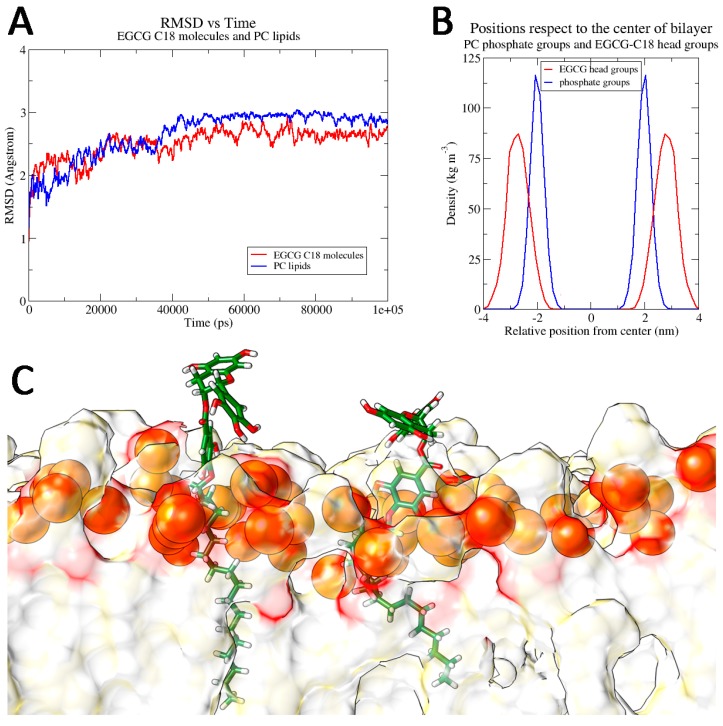
(**a**) Root Mean Square Deviation (RMSD) of C18-EGCG molecules and PC lipids in function of time. (**b**) Mass density profiles of C18-EGCG heads and phosphate groups respect to the center of simulation box. (**c**) C18-EGCG organization inside liposome vector. Results obtained from Molecular Dynamic Simulations.

As previously reported, even though the EGCG structure enables it to interact with the the polar heads of phospholipids present in the bilayer [15,51], only part of EGCG molecules is efficiently bound to the lipid mambrane. Part of EGCG molecules lies in the water phase, thus able to react with the radicals generated from AAPH decomposition, preventing them to interact with membrane lipids. Furthermore, since the cathechin does not efficiently enter within the membrane, it is not able to diffuse into the liposome core in order to act as ‘‘chain breaker’’ and stop the propagation of lipid peroxidation there. The introduction of a lipophilic chain in C18-EGCG can significatively alter its behavior. Thus, in order to gain structural insights, molecular dynamics (MD) simulations have been employed to study at a molecular scale the interaction of C18-EGCG with a PC membrane systems, and the results are summarized in Figure 5. The system reached its stability after 70 ns of MD simulation, as confirmed by RMSD values (Figure 5a), and showed a very similar stabilization pathway for both C18-EGCG molecules and PC lipids. In fact, considering the last 30 ns of MD simulation (after reaching the steady state), C18-EGCG molecules showed very similar RMSD values (2.7 ± 0.2 nm) with respect to those obtained for PC lipids (2.8 ± 0.1 nm), meaning that catechin molecules become an integral part of the PC membrane without affecting the structural properties of the lipid system. After testing the effective stability of the mixed composition membrane, the orientation of the aromatic heads of C18-EGCG along MD trajectories was investigated. Only the last 30 ns of MD simulation have been considered for this analysis, in order to identify the behaviour of C18-EGCG molecules after having reached membrane stability. Analysing the mass density profiles of the phosphate groups of PC lipids and the aromatic heads of C18-EGCG molecules (Figure 5b), we found that these last expose their aromatic moyeties in the aqueous phase, avoiding folding back inside the lipid phase. In addition, the gallate moiety appeared to be placed very close to the phosphate groups, on the bilayer surface, being able to intercept all radicals targeting the aliphatic chain within the lipid system (Figure 5c). This strucutral configuration could account for the increased antioxidant activity recorded in the AAPH-induced peroxidation experiments with respect to DPPH assay: the aliphatic tail allows the C18-EGCG to fit into the bilayer, hence exposing the polyphenolic portion towards the watery environment. As a consequence, this antioxidant can efficiently react with the approaching radicals from the aqueous side, but also into the bilayer because of the presence of the gallate moiety within the membrane. 

### 3.6. C18-EGCG Protective Efficacy against Oxidative Stress Induced Cell Death

**Figure 6:**
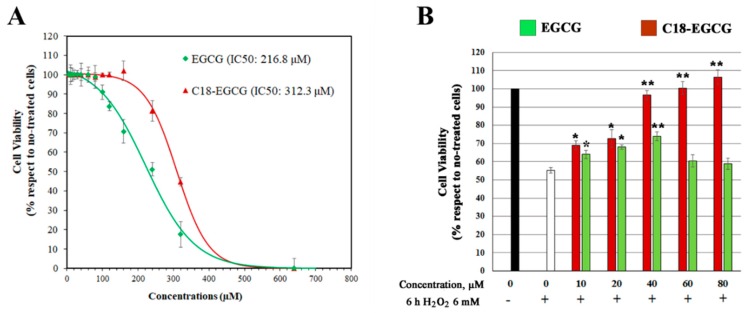
(**a**) IC50 Values for C18-EGCG and EGCG in cytotoxicity assays with retinal cell lines. ARPE-19 cells were treated with 0–640 μM concentration range of EGCG and C18-EGCG for 48 hours. Cell viability was measured by MTT assay and the values are expressed with respect to cell viability values in untreated cells normalized to 100%. Cytotoxicity curves represent 3 experiments with 5 replicates for each drug concentration. Vertical bars in the graphics represent ± SD. Relative IC50 values shown were determined by non-linear regression using the Sigma Plot Software. (**b**) Effect of C18-EGCG in ARPE19 cells after H_2_O_2_ exposure. The cells were pre-treated with increasing concentrations of C18-EGCG and EGCG for 24 h, before being exposed to 6 mM H_2_O_2_ for 6 h. Cell viability was determined by the MTT assay. Data are reportyed as means ± S.D. of five independent experiments, each performed in triplicate. ** *p* < 0.001, * *p* < 0.05 difference from control, representing H_2_O_2_-exposed cells, as reported in the figure.

EGCG and the newly synthesized C18-EGCG was firstly screened for cytotoxicity against ARPE19 cell line at different concentrations and the the IC50 (50% growth inhibition) has been determined by the MTT assay. Both derivatives inhibited cell proliferation (Figure 6a); the non-toxic EGCG behavior was found between 1 and 80 μM concentration; by increasing this value, a decrease in cells growth was observed with an IC50 of 222.1 μM; interestingly, the C18-EGCG was about 1.5-fold less toxic, with an IC50 of 312.3 μM. The different behaviour could be attributed to EGCG tendency to induce aggregation [47] and lysis of membranes, acting as detergent-like species [49] but also to a more pronounced pro-oxidative effect of EGCG respect to C18-EGCG. The characteristics of polyphenols to possess both antioxidant as well as prooxidant properties [52] is infact well known; they can act as double edge sword and generate ROS as result of their auto-oxidation. It has been reported that EGCG is not stable and can be oxidized on both the B and the D ring. The autoxidation of EGCG further leads to the formation of H_2_O_2_ and other reactive oxygen species [53]. Because the mechanism for the autoxidation of EGCG is based on the formation of its quinone form, the presence of the aliphatic tail in 4” position of C18-EGCG, is responsible of a minor DPPH radical scavenging, but at the same time contributes to diminish the polyphenol toxicity due to the autooxidation process [54].

Based on these results we choose the 10–80 µM dose range to study the C18-EGCG potential ability to protect ARPE-19 cells from oxidative damage mediated by hydrogen peroxide [55].

After pre-treatment of ARPE-19 with EGCG and C18-EGCG for 24 h, two washing steps were performed in order to eliminate the derivative molecules not able to enter within cells and/or to strongly interact with lipid bilayer before the subsequent treatment with hydrogen peroxide. The contribution of EGCG to the protection against the H_2_O_2_ consequences was dose-dependent, with the best result obtained at 40 µM concentrations (ca. 20% more cell viability than H_2_O_2_-exposed cells) (Figure 6b). At the same concentration, the functionalization carried out upon EGCG, markedly enhanced the protective effect against oxidative stress with an increase in cell survival of 41% compared to H_2_O_2_-exposed group cells (*p* < 0.001). It is interesting to observe that EGCG protective effect is particularly reduced for concentration higher than 40 µM while C18-EGCG maintains its protective efficacy (ca. 43%–45% more cell viability than H_2_O_2_-exposed cells). The different behavior can be attributed to the different lipophilicity of the two antioxidants: since no specific transporters of polyphenols were found in mammalian cells, the biological activity of EGCG and its C18 derivative is related to the molecules passive transport across the cellular membrane which strongly depends on their lipophilicity [56]. The liposomal/buffer partitioning experiments showed that the presence of the hydrophobic carbon chain in C18-EGCG effectively increases its interaction with the lipidic moieties of lipid bilayer; therefore, after the cellular washing step, the C18-EGCG remains inserted inside cell membrane while part of the more hydrophilic EGCG, likely standing in the water phase will be brought away. As a consequence, the lipid moiety increases the antioxidant protection of the newly sinthesized derivative. The data also reflect the lower toxicity of C18-EGCG respect to EGCG, probably due to a diminished pro-oxidant activity. A worthy of note application of this new EGCG derivative is its combined use with other antioxidant compounds. As an example, it has been reported that, in virtue of their antioxidant properties, carotenoids play a role in the prevention and treatment of age-related ophthalmic diseases and can be used together with EGCG [57]; poliphenols and carotenoids can work with different mechanisms and could be interesting to employ the lipophilic C18-EGCG in synergy with lutein and study the pathways involved in their radical scavenging action. 

## 4. Conclusions

The insertion of a C18 chain in the EGCG structure allows to obtain mainly EGCG monoalkylated in 4″ position of ring D, as confirmed by ^1^H and ^13^C NMR experiments. Even though the antioxidant activity of EGCG derivative was inferior to that of EGCG in terms of DPPH radical scavenging activity, C18-EGCG presented instead an increased ability to protect retinal cells. This finding can ben ascribed to the pecular structure of C18-EGCG; the increased lipophilicity determines a higher affinity toward the cellular membrane and the presence of the gallate moiety very close to the phosphate groups allows the antioxidant to intercept radicals targeting the aliphatic chain within the lipid system.The developed combined *in silico*/experimental approach has proved to be a good tool in devolping an efficient lipophilic antioxidant and thus, it can be used to go further in rational drug design of novel compounds with improved activity and less toxicity at cellular level.

## Figures and Tables

**Table 1:** ^1^H NMR Chemical Shifts ^a^.

	EGCG	C18-EGCG
H-2	5.07 s	5.07 s
H-3	5.56 m	5.42 s
H-4a	2.91 dd (2.2, 17.4)	2.97 dd (2.4, 13.9)
H-4b	3.04 dd (4.6, 17.4)	3.03 dd (3.9, 13.9)
H-6	6.06 d (2.6)	6.03 m
H-8	6.03 d (2.6)	6.03 m
H-2′	6.64 s	6.67 s
H-6′	6.64 s	6.67 s
H-2″	7.03 s	7.05 s
H-6″	7.03 s	7.05 s
OCH_2_	-	4.09 t (5.5)

^a^ Spectra measured in acetone-d6 at 298 K. Chemical shifts are expressed in ppm downfield from the signals for TMS and coupling costants in Hertz are in parentheses.

**Table 2:** ^13^C NMR Chemical Shifts ^a^.

	EGCG	C18-EGCG
C-2	78.11	78.27
C-3	69.45	70.23
C-4	26.65	26.64
C-5	157.54	157.53
C-6	96.50	96.52
C-7	157.80	157.91
C-8	95.79	95.92
C-9	157.08	157.13
C-10	98.98	98.91
C-1′	130.71	130.53
C-2′	106.76	106.80
C-3′	145.96	146.61
C-4′	133.14	133.45
C-5′	145.96	146.62
C-6′	106.76	106.80
C-1″	121.78	126.21
C-2″	109.97	109.90
C-3″	146.30	151.39
C-4″	138.83	139.57
C-5″	146.30	151.39
C-6″	109.97	109.90
CO	166.17	166.31
OCH_2_	-	73.33

^a^ Spectra measured in acetone-d6 at 298 K. Chemical shifts are expressed in ppm downfield from the signals for TMS.

**Table 3:** EGCG anions relative energy calculated at B3LYP/6-31+G(d,p) level.

Ring	Oxyanion Position	ΔE (kJ/mol)
**D**	4O	0.00
**D**	3O	2.70
**D**	5O	12.06
**B**	4O	4.64
**B**	3O	10.45
**B**	5O	5.56
**A**	5O	6.48
**A**	7O	9.56
